# A SARS-CoV-2 outbreak associated with five air force bases and a nightclub following the lifting of COVID-19-related social restrictions, United Kingdom, July-to-September 2021

**DOI:** 10.1017/S0950268823000134

**Published:** 2023-02-03

**Authors:** Michael James Taylor, Jharna Kumbang, Kate Bamford, Hanouf Mohammed Jazuli Ismail, Phil Huntley, Natalie Liddle, Jim Errington, John Mair-Jenkins

**Affiliations:** 1 Health Protection Team, UK Health Security Agency East Midlands, Nottingham, UK; 2 Lincolnshire County Council, Lincoln, UK; 3 Royal Air Force, Sleaford, UK; 4 Field Service Midlands, UK Health Security Agency, Nottingham, UK

**Keywords:** COVID-19, outbreak management, outbreaks, prevention, SARS-CoV-2

## Abstract

We describe the management of two linked severe acute respiratory coronavirus 2 (SARS-CoV-2) outbreaks, predominantly amongst 18–35-year-olds, in a UK county in July-to-September 2021, following the lifting of national coronavirus disease 2019 (COVID-19)-associated social restrictions. One was associated with a nightclub and one with five air force bases. On week beginning 2nd August 2021, air force contact tracing teams detected 68 cases across five bases within one county; 21 (30.9%) were associated with a night-time economy venue, 13 (19.1%) with night-time economy venues in the county's main town and at least one case per base (*n* = 6, 8.8%) with a particular nightclub in this town, which itself had been associated with 302 cases in the previous week (coinciding with its reopening following a national lockdown). In response, Public Health England/United Kingdom Health Security Agency, air force and local authority teams collaboratively implemented communication strategies and enhanced access to SARS-CoV-2 testing and vaccination. Key challenges included attempting to encourage behaviours that reduce likelihood of transmission to a population who may have considered themselves at low risk from severe COVID-19. This report may inform future preparation for, and management of, easing of potential future pandemic-related social restrictions, and how an outbreak in this context may be addressed.

## Introduction

Coronavirus disease 2019 (COVID-19), caused by severe acute respiratory coronavirus 2 (SARS-CoV-2) [1], has been associated with over 660 million cases and 6.7 million deaths between its discovery in December 2019 [2] and 16th January 2023 [3]. Transmission is through respiratory droplets or aerosols that are inhaled or absorbed through mucous membranes [4]. Infection can be asymptomatic or can lead to: mild illness commonly involving cough, fever and anosmia; pneumonia; critical complications such as acute respiratory distress syndrome, thrombosis and sepsis or can be fatal [5]. Effective SARS-CoV-2 vaccinations have been developed [6, 7], but they do not confer absolute protection and the pathogen has the potential to evolve into vaccine-resistant variants [8]. Evidence relating to alternative forms of infection prevention and control (IPC), therefore, may be pertinent to the management of future potential SARS-CoV-2 outbreaks.

Non-pharmaceutical COVID-19 interventions may include social restrictions; isolation of cases and contacts; enhanced cleaning; use of personal protective equipment (PPE) and enhanced testing to increase case finding, including mass testing for asymptomatic individuals [5, 9–12]. The effectiveness of a preventative intervention may be highly dependent on regional SARS-CoV-2 community incidence and prevalence, and on characteristics of the recipient population [9, 13]. For example, an individual's age may be a strong predictor for whether they adhere to social restriction guidance: for adults in the UK, compliance with national lockdown rules appears to have been considerably lower for those aged 25–34 years than for older age groups [14]. Appropriately tailoring COVID-19 prevention interventions to have maximum impact upon a target population may increase the likelihood of their effectiveness, but there is a paucity of evidence relating to this [12].

SARS-CoV-2 transmission risk is highest in confined and unventilated spaces, where there is close contact with others, and in crowded places [4] and accordingly, nightclubs are a context in which infection rates can be particularly high [15, 16]. This was reflected in UK public health policy with nightclubs being required to close at the start of the first national COVID-19 lockdown on 26th March 2020, and only reopening when all national COVID-19 social restrictions were lifted on 19th July 2021 [17–19].

Previous large COVID-19 outbreaks amongst populations residing in semi-closed institutions have been linked to clusters of local night-time economy venues [20]. Here we describe our experiences of addressing two connected outbreaks that took place in July–September 2021: one associated with five air force bases, and one associated with a nightclub that had recently reopened. The affected population predominantly consisted of adults aged 18–35 years, whose age range indicated them unlikely to be at significant personal risk from the disease [21, 22], but also to be less likely than older groups to have received two SARS-CoV-2 vaccinations [23]. The high number of cases associated with these settings meant there was a risk of occurrence of an outbreak occurring in the wider community affecting more vulnerable individuals.

The final easing of all restrictions associated with the UK's third national lockdown, including the reopening of nightclubs, took place on 19th July 2021 [17–19]. Overall UK SARS-CoV-2 COVID-19 case rates saw a decline during the latter half of July 2021; possibly due to a combination of warm weather causing less indoor socialising, increased COVID-19 app self-isolation alerts, decreased testing (e.g. due to some schools closing for summer holidays) or a relative decrease in incidence following a peak associated with the Euro 2020 football tournament (delayed until 2021 due to the COVID-19 pandemic) [24]. Infection rates in the UK county where the outbreaks described, however, increased in late July 2021, particularly amongst adults aged under 30 years [25]. Although there are reports of COVID-19 outbreaks following the reopening of schools [26], and studies of area-level incidence during the reopening of businesses and social venues [27], we are not aware of any published reports of outbreaks occurring directly after widespread reopening of businesses or social venues.

## Objectives

The primary objectives of describing these outbreaks and the responses are to inform future decisions on how to manage easing of potential future social restrictions associated with communicable disease epidemics (by illustrating possible unintended consequences of easing restrictions suddenly) and how an outbreak in such a context may be addressed. A secondary objective is to describe a multicomponent outbreak management approach that was adopted for a population for whom many were likely to perceive their personal risk from the disease to be low.

### Outbreak detection

PHE/UKHSA (Public Health England Health Protection, which transitioned into United Kingdom Health Security Agency in October 2021) noted a 60% increase in SARS-CoV-2 reported cases, from 158 to 253, over 2 days in two local government districts of a UK county – that of the county's main town and the district bordering this town to the south – on 30 July 2021. Most cases were aged 20–29 years, and many were associated with a particular nightclub in the main town. Notifications from regional surveillance on 31st July indicated 144 cases to have been associated with this nightclub since 19th July. This report also indicated a recent increase in case rates associated with five air force bases, also located in the county, which had been associated with 205 cases over the previous 28 days (see Table 1 for the distances between each base and the nightclub in the county main town; base 2, base 3 and base 5 were located in the above-mentioned district to the south of the main town). Reports from air force contact tracing teams indicated some of these cases to be linked to the nightclub.

**Table 1.** Population, case numbers and distance from the nightclub of air force bases in the county

Name of base	Total number of personnel and civilians that may be present on base at one time	Cases between 2nd August and 12th September (air force contact tracing data)	Proportion of total (attack rate) (%)	Direct distance (‘as the crow flies’ from nightclub (kilometres)
Air Force Base 1	Between 2500 and 4000	53	1.3–2.1	28.7
Air Force Base 2	Around 2000	33	1.7	21.8
Air Force Base 3	Between 300 and 500	37	7.4–12.3	16.5
Air Force Base 4	Around 600	16	2.7	7.8
Air Force Base 5	Around 2000	68	3.4	6.5

## Methods

### Study design

A rapid outbreak investigation using a retrospective observational design, with descriptive epidemiological analyses.

### Epidemiological investigation

#### Case definition

COVID-19 symptomatic and asymptomatic individuals in the UK county where the outbreaks took place who received a positive SARS-CoV-2 polymerase chain reaction (PCR) test result between 19th July and 12th September 2021 following an exposure in the UK county at the nightclub or one of the five air force bases.

#### Data collection

PHE/UKHSA regional surveillance, from enhanced contact tracing information gathered from those who received a positive PCR result, is described. No individually identifiable information is provided. UKHSA/PHE contact tracing and test data were used to generate ‘common exposure reports’ which identified individuals who had been at one of the five air force bases, or the nightclub under investigation, who had received a positive PCR result. We included both those who might have transmitted SARS-CoV-2 to others, and those who could have been infected with SARS-CoV-2 at the sites. The assumed infectious period was between 2 days prior, and 10 days post, the earliest of the symptom onset date and the first-positive test date (or the first-positive test date if symptom onset data were not available); and the assumed incubation period was between 7 and 3 days prior to this date. The information in this dataset comprises information entered by the affected individual tested on the test request form, with supplementary information having been added by contact tracers who phoned the individual. In addition, after the increase in air force base cases was noted at the end of July, we received weekly updates from air force contact tracing teams on the number of new cases that contact tracing teams at the bases had detected and the category of night-time economy exposures. The three categories were (a) at least one night-time economy venue (a pub, bar or nightclub), outside, but not within, the county's main town; (b) at least one night-time economy venue in the county's main town, but not the nightclub or (c) the nightclub (which is in the county's main town), with or without visits to other night-time economy venues.

## Results

### Regional context

The number of new SARS-CoV-2 cases per day (7-day rolling average) in the local county was 341 (0.197% per unit population) on 19th July 2021, rose to a peak of 402 (0.233%) on 5th August, then gradually reduced to 298 (0.173%) on 12th September [25].

### Nightclub outbreak

**Fig. 1.**
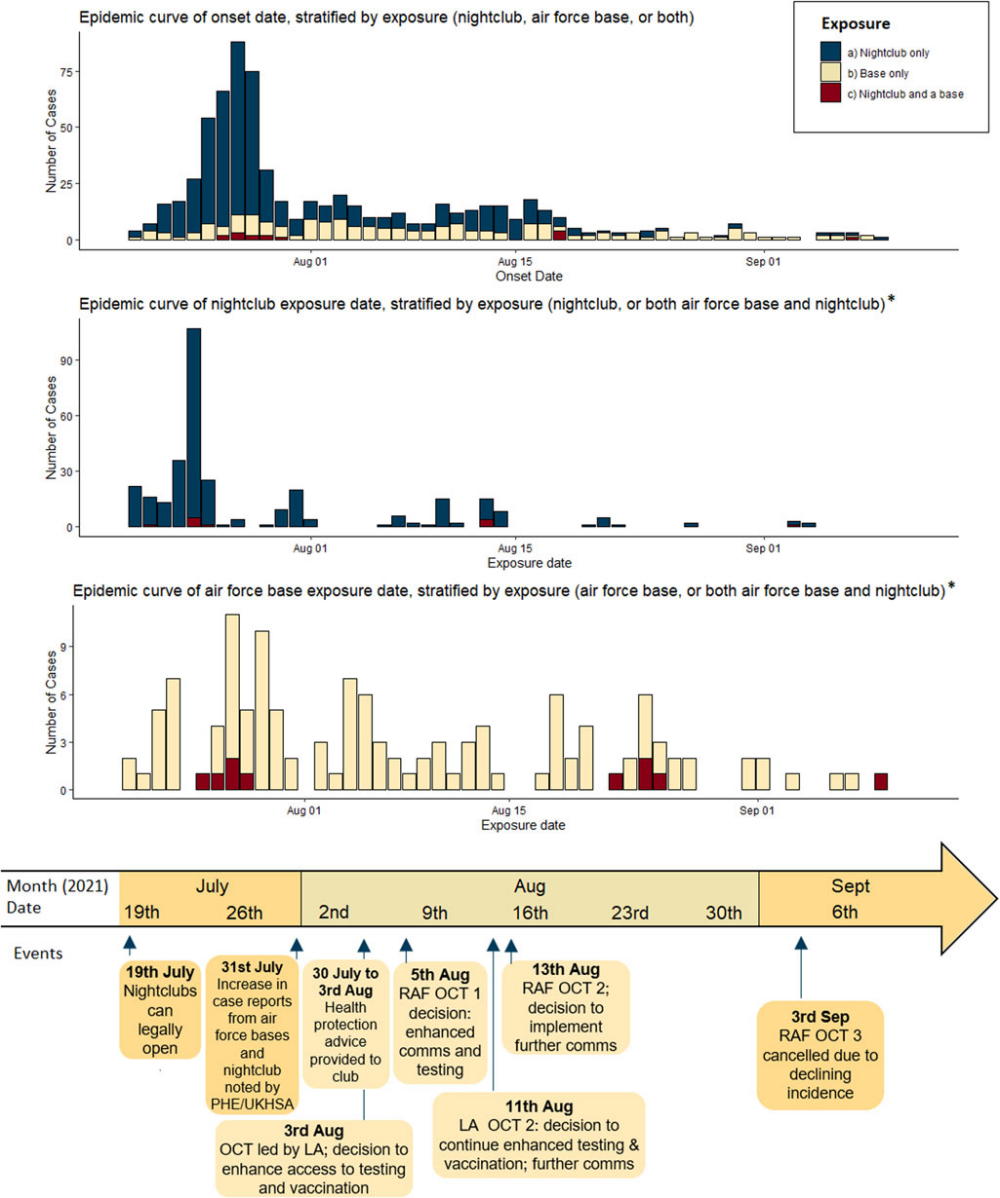
Epidemic curves of nightclub and air force bases and timeline of events.

Cases associated with the nightclub with test dates between 19th July and 12th September numbered 515, with 302 being on week ending 1st August (see Table 2 and Fig. 1). Of these cases, 432 (84.5%) were under the age of 25 years and 362 reported a home postcode in the local county, of which 128 were in the county's main town. Fifteen reported working at one of the five air force bases. The date associated with the highest number of exposures by far were Saturday, 24th July, which was associated with 107 exposures, representing 30.7% of those associated with the nightclub (the second highest was 36 (10.7%) on Friday, 23rd July).

**Table 2.** Cases per week by test date identified through PHE/UKHSA common exposure reports or air force contact tracing data

	Base 1	Base 2	Base 3	Base 4	Base 5	Total RAF base cases	Total nightclub cases	Nightclub and a base
Age (median, interquartile range)	29.5 (25.8–36.0)	26.0 (23.8–39.3)	29.5 (22.3–34.0)	34.0 (23.0–52.0)	33.0 (27.8–42.0)	30.0 (25.0–38.5)	20.0 (19.0–23.0)	24.0 (21.0–28.0)
*n* female (%)	5 (6.6%)	7 (19.4%)	11 (32.4%)	3 (27.3%)	14 (21.9%)	39 (17.8%)	241 (46.8%)	1 (6.7%)
Number of cases^a^								
19th–25th July	9	5	3	0	7	24	44	0
26th July–1st Aug	22^a^	8	5	3	18^a^	55	302	10
2nd–8th Aug	12	7^a^	9	3	13^a^	43	57	0
9th–15th Aug	11	1	8	1	9	30	47	0
16th–22nd Aug	7	6	4	3	7	17	46	1
23rd–29th Aug	7	4	5	0	3	19	10	3
30th Aug–5th Sep	6	3	0	1	4	14	4	0
6th–12th Sep	2	2	0	0	3	7	5	1
Total	76	36	34	11	64	219	515	15

a A case this week had an exposure associated with two bases and is counted once each amongst the counts for these bases, and once overall for all five bases.

### Air force bases outbreak

UKHSA data identified 214 cases with a test date between 19th July and 12th September who were living, working or had visited at least one of the five air force bases in the county (two reported to have visited two bases). See Table 2 for the age and gender distribution of cases. Air force contact tracing teams identified 207 cases with test date between 2nd August and 16th September; over this time, 47 (22.7%) were associated with a night-time economy venue, 24 (11.6%) with a night-time economy venue in the county's main town and 12 (5.8%) with a particular nightclub in this town (air force contact tracing data are summarised in Supplementary materials S1–S3).

**Fig. 2.**
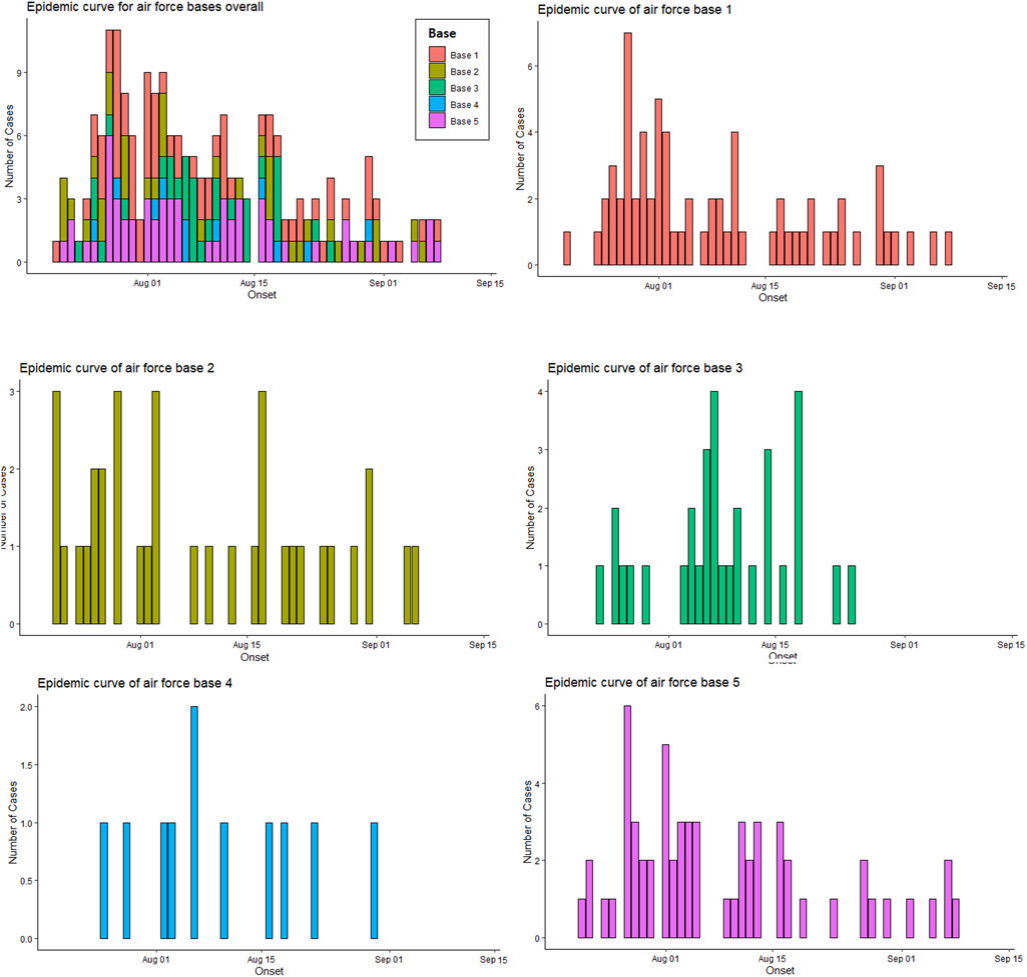
Epidemic curves of the air force bases overall, and each base individually.

The number of cases per week associated with the air force bases peaked on week ending 1st August – the same week as that in which the peak in case reports associated with the nightclub occurred. Between 2nd August and 12th September, the number of weekly cases reduced across all five bases and the proportion of cases associated with the night-time economy reduced (see Figs 1 and 2 and Supplementary materials S1–S3). Exposure dates for the bases were more evenly distributed than those of the nightclub; the date associated with the highest number of exposures was 27th July (11 (7.9%)).

### Cases associated with an air force base and the nightclub

Fifteen cases were identified as being associated with both an air force base and the nightclub. Dates of symptom onset, test date and activity date indicated that 12 may have contracted COVID-19 at the nightclub (and were unlikely to have been infectious when they visited), while the remaining three may have contracted COVID-19 prior to the nightclub visit and may have been infectious during their visit. Two thirds (10) of these cases had a test date between 26th July and 1st August (see Table 2), and three may have contracted COVID-19 on 24th July – the date associated with the highest number of exposures at the nightclub.

### Outbreak control measures

#### Nightclub outbreak

Between 30th July and 3rd August 2021, local authority health protection team members conducted risk assessments with, and visits to, the affected nightclub and reviewed workplace processes. This led to the nightclub organising daily IPC briefings to staff; enhanced touchpoint cleaning and greater compliance with face mask guidance. The nightclub reportedly had relatively low ventilation; a capacity of 1800; was particularly busy during the weekend following its reopening and often had numerous attendees from outside the city (much of the county is semi-rural, and many travel to attend night-time economy venues in the main town).

**Fig. 3.**
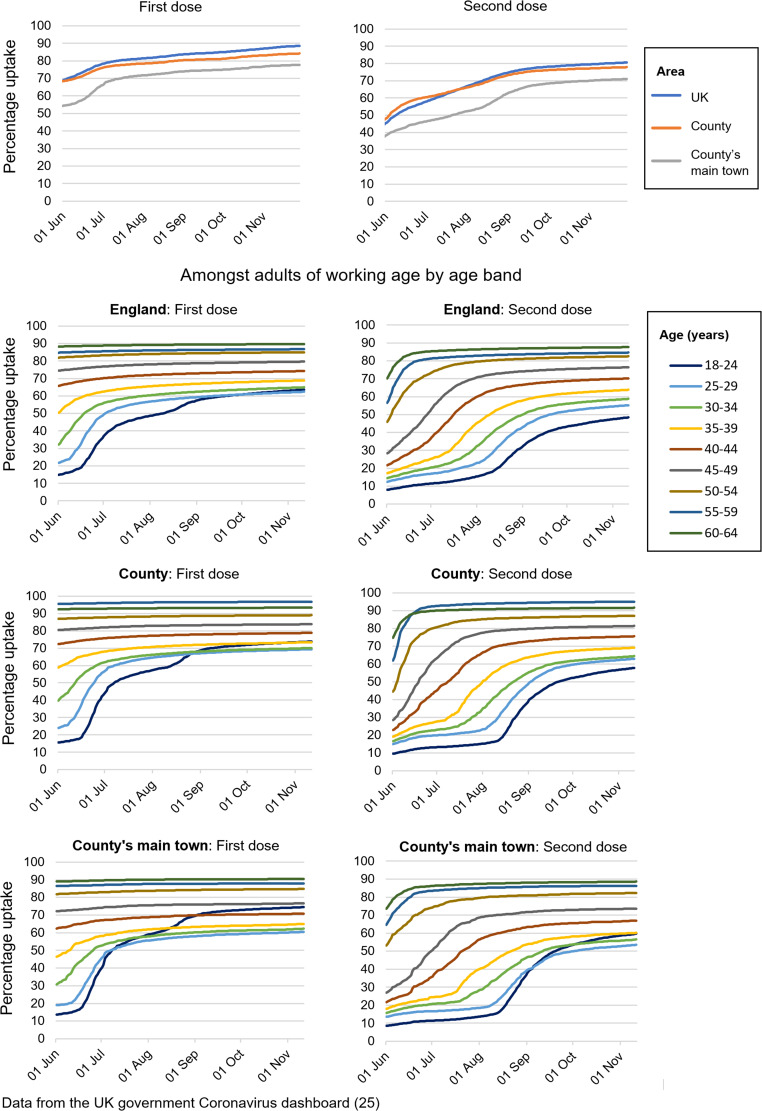
Vaccination uptake (cumulative) in UK, England, the county in which the outbreaks took place, and the county's main town.

On 3rd August, an outbreak control team (OCT) meeting, led by the local authority, with support from PHE/UKHSA, was held. As of 1st August 2021, the proportion of the population of the county's main town who had received two SARS-CoV-2 vaccinations (51.4%) was lower than that of the average for the county (65.5%) and the UK (66.8%) [25] (see Fig. 3). Agreed outbreak management measures, therefore, included plans to improve access to testing and vaccination, especially for adults aged 18–35 who represented the majority of outbreak-associated cases. This involved arranging for an agile testing unit to be based near city areas associated with the highest number of COVID-19 cases, a pop-up vaccination hub to be placed alongside this testing unit, and city customer-facing businesses (especially those visited by young adults) to provide lateral flow tests to customers.

Plans were also made to work with environmental health teams to assess COVID-19 IPC practices at night-time economy businesses and provide related advice. A health promotion strategy was developed, which involved collaboration with city night-time economy businesses to enhance promotion and provision of SARS-CoV-2 tests and vaccinations, and an outreach team engaging with the local 18–30-year-old population, providing them with test kits and signposting them to vaccination centres.

The local authority also developed a communications campaign aiming to encourage adults younger than 30 years who attend night-time economy venues to adopt behaviours that reduce the risk of SARS-CoV-2 transmission. This campaign involved creation and dissemination of health promotion images for posters, postcards and social media posts advising that while socialising, making space and avoiding crowds helps to reduce risk of transmission, as does regular testing. These recommendations were also promoted at interviews with local radio stations, by the nightclub in their promotional materials, by taxi firms (via text message to people booking evening taxi journeys) and to schools ahead of anticipated exam results celebrations.

#### Air force bases outbreak

An OCT meeting on 5th August 2021 was led by PHE/UKHSA and supported by the air force, local authority and national partners including Department of Health and Social Care and NHS England and Improvement. Potential transmission pathways for the cases and risks of ongoing transmission were identified. In addition to the night-time economy, these included staff working across multiple bases and the bases consisting of both people who lived there and people who lived elsewhere and commuted daily.

Plans were made to improve access to testing for all employees and residents at the air force bases, including non-service personnel. Agreed actions also included cascading of communications promoting vaccination and recommending an 8-week, rather than 12-week, gap between first- and second-vaccination doses as per recently updated UK policy [28]. Air force, local authority partners and PHE/UKHSA collaboratively produced and circulated messages emphasising the importance taking personal responsibility to reduce risk of SARS-CoV-2 transmission and to increase testing and vaccination uptake. Materials from the above-described communication campaign developed by the local authority were also disseminated within the bases. The multiagency response team continued to collaborate and Figure 1 summarises subsequent relevant OCT meetings. On 19th October 2021, it was noted that case rates had been decreasing for several weeks, with no evidence of any substantial increase in case numbers at any of the bases in the past 28 days.

#### Vaccination uptake

There was a considerable increase in vaccination uptake amongst young adults, especially those aged 18–24, between 5th August and 12th September (see Fig. 3) in the county and the county's main town. For age categories between 18 and 34 years, second dose uptake increased by 21.2% across England (to 46.3%), compared to 27.3% in the county (to 53.2%) and 27.4% in the county's main town (to 47.3%).

## Discussion

We describe two linked COVID-19 outbreaks that occurred in a UK county in in July-to-September 2021: the first was associated with a nightclub and the second was associated with five air force bases. Both outbreaks predominantly affected 18–35-year-olds. The outbreaks occurred in the weeks following the final restrictions being lifted after the UK's third national lockdown during the COVID-19 pandemic. The affected population – young adults – represented a group at relatively low risk of developing severe SARS-CoV-2 infection complications [21, 22], and therefore they were also less likely than older adults to have received COVID-19 vaccinations due to older age groups being prioritised for vaccination [23]. It was noted that the nightclub, situated in the county's main town, was particularly busy on the weekend following restrictions being lifted, and many had travelled from outside of the town to attend, which may have increased the risk of high transmission rates. Our analysis of the timing of exposures suggests that the outbreak at the nightclub and those at the bases were likely to have propagated one another to an extent, but that the nightclub outbreak likely led to a greater increase in cases at the bases than vice versa. Base 3 had by far the highest attack rate of the five bases. We cannot be certain of the reasons for this, but of bases with a median case age under 30 years, it was the closest geographically to the nightclub. It might, therefore, have been associated with relatively high levels of nightclub attendance, leading to increased COVID-19 incidence amongst a group who were young and hence relatively unlikely to be fully vaccinated [25].

As of July–August 2021, the B.1.617.2 (‘Delta’) variant was dominant in the UK [29]. Although all mandated social restrictions had eased on 19th July, there remained a legal requirement to self-isolate for those who tested positive for COVID-19 for 10 days following onset of symptoms or, if asymptomatic, from the date of the positive test result [30]. Asymptomatic contacts of cases were also legally required to isolate for 10 days from the time of contact with the case [30]. At this time, while most of the general UK population had received two SARS-CoV-2 vaccination doses, over a third of adults under the age of 35 years were yet to receive a single dose [25] (see Fig. 3). Therefore, public health measures that were adopted to control the outbreak focused on health promotion (specifically of vaccination, testing and avoiding close contact and crowds where possible); working with organisations to ensure safe practices; and improving community access to testing and vaccination. Many attendees of the club resided in the county's main town, which was noted to have a relatively low vaccination uptake; this was likely to be largely due to its consisting of high numbers of young adults [31] who were less likely to be in high-priority vaccination groups [23]. Although this population represented a group with few at high risk of severe disease [21, 22], the low vaccination uptake meant that an outbreak was more likely to occur [32].

On 19th July 2021, it became legal to attend nightclubs in the UK [17–19] and our communication strategies needed to consider that the main target population (young adults) were: not acting illegally; not necessarily at high personal risk from serious COVID-19 illness and may have felt they had already made considerable personal sacrifices for others during the previous 16 months of the pandemic [17]. The communication campaign accordingly focused on personal responsibility (the message conveyed stated that ‘COVID-19 is still here’) but rather than dissuade the messages' target audience from going out at all, made the less demanding request to ‘Make space. Avoid crowds. Keep testing’. The target audience may have also been more likely to be motivated to comply with this than a demand to desist socialising altogether [33, 34]. Previous evidence has suggested that people may become more motived to perform behaviours that reduce transmission risk when reminded of the responsibility to protect others who are more vulnerable [35].

The multiagency collaboration within our outbreak response team enabled a coordinated and efficient delivery of multiple interventions. When it became apparent that the two outbreaks were linked and affected similar population demographics, this indicated potential appropriateness of the targeted communications campaign that had been developed by the local authority for use at air force bases. Collaborative surveillance and monitoring of contact tracing data between PHE/UKHSA and the air force was helpful in ensuring validity of incidence and exposure, which was useful for monitoring the ongoing situation. The presence of multiple agencies at OCT meetings facilitated the sharing of intelligence, preventative strategy components and the coordination of actions for respective organisations.

Study limitations include that we do not have complete information on the number of people exposed during the described outbreaks. Furthermore, due to the high levels COVID-19 incidence in the region at the time of data collection, some of the cases presented here as being associated with the air force bases, the nightclub or both may have contracted COVID-19 due to an alternative exposure. We believe, however, that the propagation patterns displayed by the outbreak epi-curves suggest that a considerable proportion of the cases presented resulted from transmission at these settings. On inspection of national contact tracing and cases data, some case variables were missing or contained errors; such errors in the wider dataset may have prevented some relevant cases from coming to our attention. A further limitation is that some of the outbreak interventions may have had beneficial effects that lasted beyond the present outbreaks that are not described here. Also, as our intervention involved the simultaneous implementation of multiple components, we are unable to provide evidence of which of the components was the most effective. It may also be possible that effectiveness was due to there being a particular combination of components, and indeed, ‘packages’ of intervention are often found to be particularly effective forms of COVID-19 prevention [9]. There is some indication, however, to suggest that efforts to promote vaccination uptake amongst young adults may have been particularly effective, as this increased considerably during our investigation, by a greater proportion than the national average, especially for 18–24-year-olds (see Fig. 3).

Previous research has indicated military bases as an example of a congregate setting where there is risk of large COVID-19 outbreaks occurring [36]. Our findings are consistent with evidence of the potential effectiveness of non-pharmaceutical outbreak–control interventions within [37] and outside such settings, including social restriction, testing, contact tracing, isolation, vaccination, hygiene and cleaning, PPE [9, 10, 12] and communicating advice relating to these strategies [35, 38]. Effective communications may involve population-wide health promotion using materials such as posters or online materials [35], or work-based risk assessments and advice [38]. Strategies such as outreach teams [39] or patient and public involvement [40] may have improved engagement with local populations and have led to a greater impact of outbreak-related messaging.

## Conclusions

To our knowledge, this is the first report to describe an outbreak affecting a business or social venue in the context of the easing of nationally mandated communicable disease-related social restrictions. It illustrates the potential for large outbreaks to occur in this context and highlights the importance of considering the risks associated with widespread reopening of social venues, especially when these may attract well attended celebrations. In the potential event of the easing of future lockdown restrictions, we recommend that policymakers consider how increases in large social gathering events may lead to high rates of transmission, and how the risk of associated outbreaks occurring could be mitigated. For example, rather than having a particular time when all large night-time economy venues can simultaneously legally open at full capacity, the reopening of such venues could be staggered, or venue capacity restrictions could be initially mandated, then incrementally eased. If this had been done at the time of the present outbreaks, particularly on the weekend following nightclubs reopening, then case numbers would likely have been far lower.

We believe our report is also novel in illustrating an example of how large outbreaks in the context of the easing of social restrictions may be addressed. The outbreaks described here primarily affected those likely to initially return to social venues following their reopening: young adults [27], who were likely at low personal risk from severe infection [21, 22]. This population may have been less motivated, compared to a more vulnerable population, to change their behaviours to reduce transmission or infection risk. It was important, however, to control the outbreak to lower the risk to the population, especially those more vulnerable to severe COVID-19. A multiagency outbreak control response was implemented, which involved enhancing access to testing and vaccination; increasing engagement with local workplaces and the public and delivering a communications campaign to encourage behaviours that reduce disease transmission. Following this multicomponent intervention package there was a substantial reduction in COVID-19 cases, and an increase in vaccination rates amongst young adults.

In future similar situations in which social restrictions are eased, it may be beneficial for public health teams to anticipate there being an increased risk of significant outbreaks and to implement measures to reduce this risk. Such measures could include working with businesses due to reopen to provide IPC advice and delivering communications campaigns that inform members of the public (especially those who may be unvaccinated or more likely to attend social venues) of behaviours they can adopt that reduce chances of disease transmission.

## Data Availability

Participant permission for public sharing of data was not obtained, so supporting data are not available.

## Supplementary material

For supplementary material accompanying this paper visit https://doi.org/10.1017/S0950268823000134. click here to view supplementary material

## References

[1] Hu B, Guo H, Zhou P and Shi Z-L (2021) Characteristics of SARS-CoV-2 and COVID-19. Nature Reviews Microbiology 19, 141–154. 33024307 10.1038/s41579-020-00459-7 PMC7537588

[2] Huang C (2020) Clinical features of patients infected with 2019 novel coronavirus in Wuhan, China. The Lancet 395, 497–506. 10.1016/S0140-6736(20)30183-5 PMC7159299 31986264

[3] World Health Organisation. WHO coronavirus (COVID-19) dashboard 2023 (cited 17th January 2023). Available at https://covid19.who.int/.

[4] World Health Organisation. Coronavirus disease (COVID-19): How is it transmitted? (cited 4th August 2022). Available at https://www.who.int/news-room/questions-and-answers/item/coronavirus-disease-covid-19-how-is-it-transmitted.

[5] World Health Organization (2021) COVID-19 Clinical Management: Living Guidance, 25 January 2021. World Health Organization. https://apps.who.int/iris/handle/10665/338882.

[6] UK Health Security Agency (2021) COVID-19: The Green Book, Chapter 14a: Coronavirus (COVID-19) vaccination information for public health professionals (cited 4th August 2022). Available at https://assets.publishing.service.gov.uk/government/uploads/system/uploads/attachment_data/file/1034368/Greenbook_chapter_14a_15Nov21.pdf.

[7] Self WH (2021) Comparative effectiveness of Moderna, Pfizer-BioNTech, and Janssen (Johnson & Johnson) vaccines in preventing COVID-19 hospitalizations among adults without immunocompromising conditions – United States, March–August 2021. Morbidity and Mortality Weekly Report 70, 1337. 34555004 10.15585/mmwr.mm7038e1 PMC8459899

[8] World Health Organisation. Tracking SARS-CoV-2 variants 2021 (cited 4th August 2022). Available at https://www.who.int/en/activities/tracking-SARS-CoV-2-variants/.

[9] Talic S (2021) Effectiveness of public health measures in reducing the incidence of COVID-19, SARS-CoV-2 transmission, and COVID-19 mortality: systematic review and meta-analysis. British Medical Journal 375, e068302. 34789505 10.1136/bmj-2021-068302 PMC9423125

[10] Hellewell J (2020) Feasibility of controlling COVID-19 outbreaks by isolation of cases and contacts. The Lancet – Global Health 8, e488–e496. 32119825 10.1016/S2214-109X(20)30074-7 PMC7097845

[11] Ferguson J (2021) Validation testing to determine the sensitivity of lateral flow testing for asymptomatic SARS-CoV-2 detection in low prevalence settings: testing frequency and public health messaging is key. PLoS Biology 19, e3001216. 33914730 10.1371/journal.pbio.3001216 PMC8112643

[12] Ingram C (2021) COVID-19 prevention and control measures in workplace settings: a rapid review and meta-analysis. International Journal of Environmental Research and Public Health 18, 7847. 34360142 10.3390/ijerph18157847 PMC8345343

[13] Nicola M (2020) Health policy and leadership models during the COVID-19 pandemic: a review. International Journal of Surgery 81, 122–129. 32687873 10.1016/j.ijsu.2020.07.026 PMC7366988

[14] Ganslmeier M, Van Parys J and Vlandas T (2022) Compliance with the first UK COVID-19 lockdown and the compounding effects of weather. Scientific Reports 12, 1–10. 35264649 10.1038/s41598-022-07857-2 PMC8907269

[15] Kang CR (2020) Coronavirus disease exposure and spread from nightclubs, South Korea. Emerging Infectious Diseases 26, 2499. 32633713 10.3201/eid2610.202573 PMC7510694

[16] Muller N (2021) Severe acute respiratory syndrome coronavirus 2 outbreak related to a nightclub, Germany, 2020. Emerging Infectious Diseases 27, 645. 10.3201/eid2702.204443 PMC7853558 33263514

[17] Institute for Government Analysis (2021) Timeline of UK coronavirus lockdowns, March 2020 to March 2021 (cited 4th August 2022). Available at https://www.instituteforgovernment.org.uk/sites/default/files/timeline-lockdown-web.pdf.

[18] Cabinet Office. Moving to step 4 of the roadmap 2021 (cited 4th August 2022). Available at https://www.gov.uk/government/publications/covid-19-response-summer-2021-roadmap/moving-to-step-4-of-the-roadmap.

[19] Gov.UK (2021) COVID-19 Response – Spring 2021 (Summary). Roadmap out of lockdown 2021 (cited 4th August 2022). Available at https://www.gov.uk/government/publications/covid-19-response-spring-2021/covid-19-response-spring-2021-summary#step-4---not-before-21-june.

[20] Harris JE (2022) Geospatial analysis of a COVID-19 outbreak at the University of Wisconsin–Madison: potential role of a cluster of local bars. Epidemiology & Infection 150, e76, 1–15. 10.1017/S0950268822000498 PMC9043656 35380104

[21] Clift AK (2020) Living risk prediction algorithm (QCOVID) for risk of hospital admission and mortality from coronavirus 19 in adults: national derivation and validation cohort study. British Medical Journal 371, m3731. 33082154 10.1136/bmj.m3731 PMC7574532

[22] Williamson EJ (2020) Factors associated with COVID-19-related death using OpenSAFELY. Nature 584, 430–436. 32640463 10.1038/s41586-020-2521-4 PMC7611074

[23] Public Health England. COVID-19 vaccination first phase priority groups 2021 (cited 4th August 2022). Available at https://www.gov.uk/government/publications/covid-19-vaccination-care-home-and-healthcare-settings-posters/covid-19-vaccination-first-phase-priority-groups.

[24] Ball P (2021) Surprise dip in UK COVID cases baffles researchers. Nature, 576, 175–176. 10.1038/d41586-021-02125-1 34345038

[25] Gov.UK. Coronavirus (COVID-19) in the UK (cited 4th August 2022). Available at https://coronavirus.data.gov.uk/?_ga=2.146722108.944676622.1601217085-1562710827.1594116739.

[26] Stein-Zamir C (2020) A large COVID-19 outbreak in a high school 10 days after schools’ reopening, Israel, May 2020. EuroSurveillance 25, 2001352. 32720636 10.2807/1560-7917.ES.2020.25.29.2001352 PMC7384285

[27] Le TD (2021) Influences of reopening businesses and social venues: COVID-19 incidence rate in East Texas county. Epidemiology & Infection 149, e28, 1–9. 10.1017/S0950268821000121 PMC7853750 33455588

[28] Department of Health and Social Care. Most vulnerable offered second dose of COVID-19 vaccine earlier to help protect against variants 2021. Available at https://www.gov.uk/government/news/most-vulnerable-offered-second-dose-of-covid-19-vaccine-earlier-to-help-protect-against-variants.

[29] Pouwels KB (2021) Effect of Delta variant on viral burden and vaccine effectiveness against new SARS-CoV-2 infections in the UK. Nature Medicine 27, 2127–2135. 10.1038/s41591-021-01548-7 PMC8674129 34650248

[30] GOV.UK (2022) Self-isolation for those with COVID-19 can end after 5 full days following 2 negative LFD tests. From Monday 17 January, people with COVID-19 in England can end their self-isolation after 5 full days, as long as they test negative on day 5 and day 6 (cited 29th December 2022). Available at https://www.gov.uk/government/news/self-isolation-for-those-with-covid-19-can-end-after-five-full-days-following-two-negative-lfd-tests.

[31] Office for Health Improvement and Disparities. Public health profiles (cited 10th August 2022). Available at https://fingertips.phe.org.uk/.

[32] Chen Y-T (2021) The effect of vaccination rates on the infection of COVID-19 under the vaccination rate below the herd immunity threshold. International Journal of Environmental Research and Public Health 18, 7491. 34299942 10.3390/ijerph18147491 PMC8305789

[33] Michie S, Atkins L and West R (2014) The Behaviour Change Wheel. A Guide to Designing Interventions, 1st Edn. Great Britain: Silverback Publishing, pp. 1003–1010.

[34] West R and Michie S (2020) A brief introduction to the COM-B model of behaviour and the PRIME theory of motivation [v1]. Qeios.

[35] Lunn PD (2020) Motivating social distancing during the COVID-19 pandemic: an online experiment. Social Science & Medicine 265, 113478. 33162198 10.1016/j.socscimed.2020.113478

[36] Marcus JE (2021) Risk factors associated with COVID-19 transmission among US air force trainees in a congregate setting. JAMA Network Open 4, e210202. 33630090 10.1001/jamanetworkopen.2021.0202 PMC7907953

[37] Marcus JE (2020) COVID-19 monitoring and response among US air force basic military trainees – Texas, March–April 2020. Morbidity and Mortality Weekly Report 69, 685. 32497031 10.15585/mmwr.mm6922e2 PMC7315849

[38] Cook TM and El-Boghdadly K (2020) COVID-19 risk tools should incorporate assessment of working environment risk and its mitigation. EClinicalMedicine 28, 100613. 33173855 10.1016/j.eclinm.2020.100613 PMC7646368

[39] Denberg TD (2008) Improving patient care through health-promotion outreach. The Journal of Ambulatory Care Management 31, 76–87. 18162801 10.1097/01.JAC.0000304102.30496.49

[40] Brett J (2014) A systematic review of the impact of patient and public involvement on service users, researchers and communities. The Patient: Patient-Centered Outcomes Research 7, 387–95. 25034612 10.1007/s40271-014-0065-0

