# Supplementary material for: A SARS-CoV-2 outbreak associated with five air force bases and a nightclub following the lifting of COVID-19-related social restrictions, United Kingdom, July-to-September 2021
Source: Epidemiol Infect. 2023 Feb 3;151:e26. doi: 10.1017/S0950268823000134 (PMC9947034; doi:10.1017/S0950268823000134)

Supplementary Materials S1: Air force base contact tracing data

Dates and exposure (% of cases per week [by test date])	Base 1	Base 2	Base 3	Base 4 and Base 5	Total
2nd to 8th Aug					
• All cases	23	11	6	28	68
• Night-time economy venue* outside the county's main town	3 (13.0%)	2 (18.2%)	3 (50.0%)	0 (0.0%)	8 (11.8%)
• Night-time economy venue in the county's main town other than the nightclub	1 (4.3%)	0 (0.0%)	0 (0.0%)	6 (21.4%)	7 (10.3%)
• The nightclub (which is in the county's main town)	1 (4.3%)	3 (27.3%)	1 (16.7%)	1 (3.6%)	6 (8.8%)
9th to 15th Aug					
• All cases	11	0	9	7	27
• Night-time economy venue* outside the county's main town	3 (27.3%)	0 (0.0%)	3 (33.3%)	0 (0.0%)	6 (22.2%)

• Night-time economy venue in the county's main town other than the nightclub	2 (18.2%)	0 (0.0%)	0 (0.0%)	0 (0.0%)	2 (7.4%)
• The nightclub (which is in the county's main town)	0 (0.0%)	0 (0.0%)	1 (11.1%)	1 (14.3%)	2 (7.4%)
16th to 22nd Aug					
• All cases	7	7	7	17	38
• Night-time economy venue* outside the county's main town	0 (0.0%)	3 (42.9%)	3 (42.9%)	0 (0.0%)	6 (15.8%)
• Night-time economy venue in the county's main town other than the nightclub	0 (0.0%)	0 (0.0%)	1 (14.3%)	1 (5.9%)	2 (5.3%)
• The nightclub (which is in the county's main town)	0 (0.0%)	0 (0.0%)	0 (0.0%)	0 (0.0%)	0 (0.0%)
23rd to 29th Aug					
• All cases	5	8	11	12	36
• Night-time economy venue* outside the county's main town	0 (0.0%)	1 (12.5%)	0 (0.0%)	0 (0.0%)	1 (2.8%)

• Night-time economy venue in the county's main town other than the nightclub	0 (0.0%)	0 (0.0%)	0 (0.0%)	0 (0.0%)	0 (0.0%)
• The nightclub (which is in the county's main town)	0 (0.0%)	0 (0.0%)	4 (36.4%)	0 (0.0%)	4 (11.1%)
30th Aug to 5th Sep					
• All cases	3	5	2	17	27
• Night-time economy venue* outside the county's main town	1 (33.3%)	1 (20.0%)	0 (0.0%)	0 (0.0%)	2 (7.4%)
• Night-time economy venue in the county's main town other than the nightclub	0 (0.0%)	0 (0.0%)	1 (50.0%)	0 (0.0%)	1 (3.7%)
• The nightclub (which is in the county's main town)	0 (0.0%)	0 (0.0%)	0 (0.0%)	0 (0.0%)	0 (0.0%)
6th to 12th Sep					
• All cases	2	4	2	3	11
• Night-time economy venue* outside the county's main town	0 (0.0%)	0 (0.0%)	0 (0.0%)	0 (0.0%)	0 (0.0%)

• Night-time economy venue in the county's main town other than the nightclub	0 (0.0%)	0 (0.0%)	0 (0.0%)	0 (0.0%)	0 (0.0%)
• The nightclub (which is in the county's main town)	0 (0.0%)	0 (0.0%)	0 (0.0%)	0 (0.0%)	0 (0.0%)
Total (2nd Aug to 12th Sep					
• All cases	53	33	37	84	207
• Night-time economy venue* outside the county's main town	7 (13.2%)	7 (21.2%)	9 (24.3%)	0 (0.0%)	23 (11.1%)
• Night-time economy venue in the county's main town other than the nightclub	3 (5.7%)	0 (0.0%)	2 (5.4%)	7 (8.3%)	12 (5.8%)
• The nightclub (which is in the county's main town)	1 (1.9%)	3 (9.1%)	6 (16.2%)	2 (2.4%)	12 (5.8%)

*A ‘night-time economy venue’ is defined here as a pub, bar, or nightclub.

Due to the nature of the data we were able to process, we cannot rule out that a minority of cases presented here were exposed to more than one base during their incubation or infectious period.

Supplementary materials S2: Cases across five bases by base and test date (air force contact tracing data)

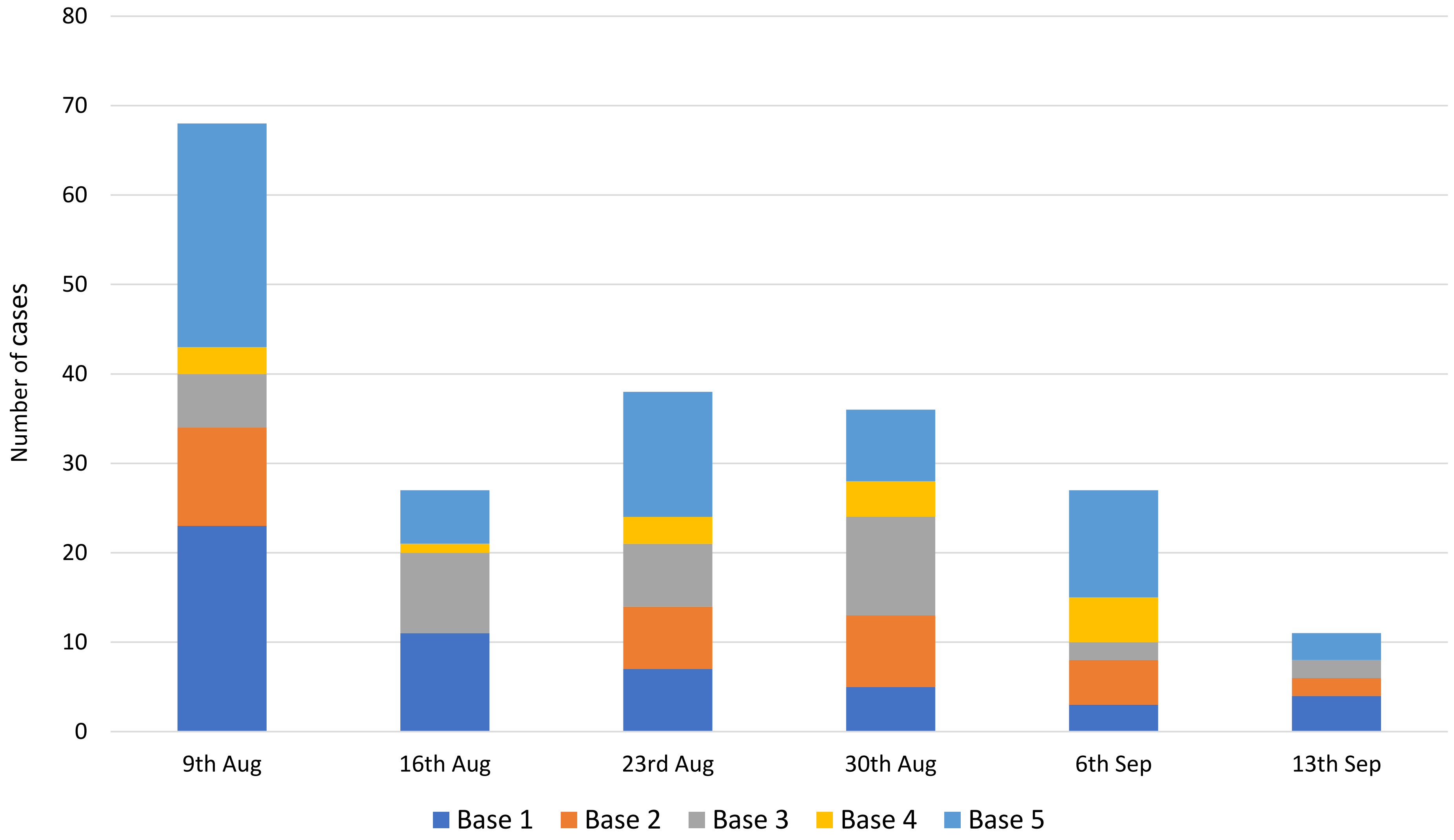

Supplementary materials S3: Cases across five bases by exposure classification and test date (air force contact tracing data)

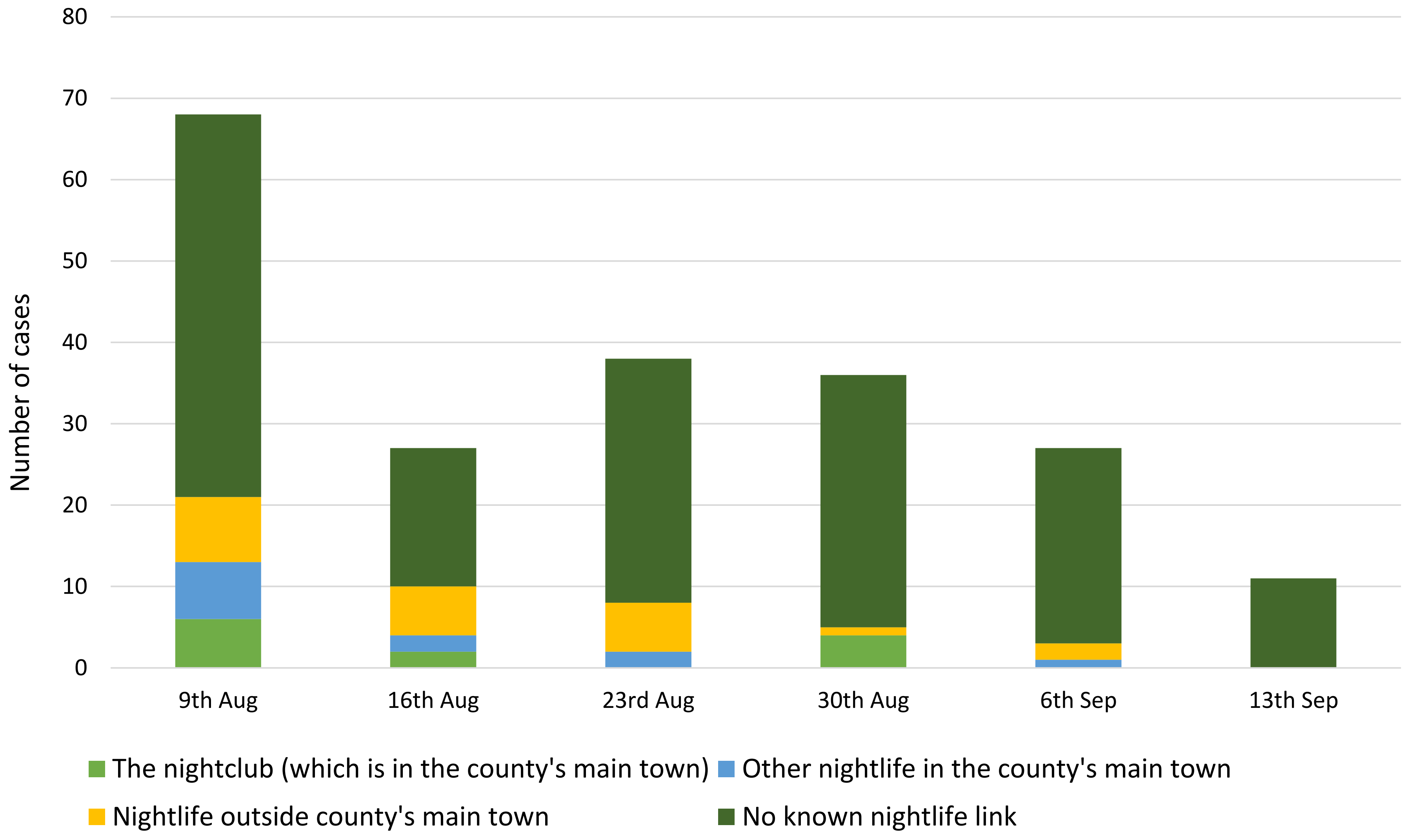

Supplement: Supplementary file 1 [file S0950268823000134sup001.pdf]
